# Comparing the use of open and closed questions for Web-based measures of the continued-influence effect

**DOI:** 10.3758/s13428-018-1066-z

**Published:** 2018-06-25

**Authors:** Saoirse Connor Desai, Stian Reimers

**Affiliations:** Department of Psychology, City, University of London, Northampton Square, London, EC1V 0HB UK

**Keywords:** Open-ended, Closed-ended, Response formats, Web-based, Misinformation, Continued influence effect

## Abstract

Open-ended questions, in which participants write or type their responses, are used in many areas of the behavioral sciences. Although effective in the lab, they are relatively untested in online experiments, and the quality of responses is largely unexplored. Closed-ended questions are easier to use online because they generally require only single key- or mouse-press responses and are less cognitively demanding, but they can bias the responses. We compared the data quality obtained using open and closed response formats using the *continued-influence effect* (CIE), in which participants read a series of statements about an unfolding event, one of which is unambiguously corrected later. Participants typically continue to refer to the corrected misinformation when making inferential statements about the event. We implemented this basic procedure online (Exp. 1A, *n* = 78), comparing standard open-ended responses to an alternative procedure using closed-ended responses (Exp. 1B, *n* = 75). Finally, we replicated these findings in a larger preregistered study (Exps. 2A and 2B, *n* = 323). We observed the CIE in all conditions: Participants continued to refer to the misinformation following a correction, and their references to the target misinformation were broadly similar in number across open- and closed-ended questions. We found that participants’ open-ended responses were relatively detailed (including an average of 75 characters for inference questions), and almost all responses attempted to address the question. The responses were faster, however, for closed-ended questions. Overall, we suggest that with caution it may be possible to use either method for gathering CIE data.

**Electronic supplementary material:**

The online version of this article (10.3758/s13428-018-1066-z) contains supplementary material, which is available to authorized users.

Over the past decade, many areas of research that have traditionally been conducted in the lab have moved to using Web-based data collection (e.g., Peer, Brandimarte, Samat, & Acquisti, 2017; Simcox & Fiez, 2014; Stewart, Chandler, & Paolacci, 2017; Wolfe, 2017). Collecting data online has many advantages for researchers, including ease and speed of participant recruitment and a broader demographic of participants, relative to lab-based students.

Part of the justification for this shift has been the finding that the data quality from Web-based studies is comparable to that obtained in the lab: The vast majority of Web-based studies have replicated existing findings (e.g., Crump, McDonnell, & Gureckis, 2013; Germine et al., 2012; Zwaan et al., 2017). However, the majority of these studies have been in areas in which participants make single key- or mouse-press responses to stimuli. Less well explored are studies using more open-ended responses, in which participants write their answers to questions. These types of question are useful for assessing recall rather than recognition and for examining spontaneous responses that are unbiased by experimenter expectations, and as such may be unavoidable for certain types of research.

There are reasons to predict that typed responses might be of lower quality for open-ended than for closed-ended questions. Among the few studies that have failed to replicate online have been those that have required high levels of attention and engagement (Crump et al., 2013), and typing is both time-consuming and more physically effortful than pointing and clicking. Relatedly, participants who respond on mobile devices might struggle to make meaningful typed responses without undue effort.

Thus, researchers who typically run their studies with open-ended questions in the lab, and who wish to move to running them online, have two options. Either they can retain the open-ended question format and hope that the online participants are at least as diligent as those in the lab, or they can use closed-ended questions in place of open-ended questions, but with the risk that participants will respond differently or draw on different memory or reasoning processes to answer the questions. We examined the relative feasibility of these two options by using the *continued-influence effect*, a paradigm that (a) is a relatively well-used memory and reasoning task, (b) has traditionally used open-ended questions, and (c) is one that we have experience with running in the lab.

## The continued-influence effect

The *continued-influence effect* of misinformation refers to the consistent finding that misinformation continues to influence people’s beliefs and reasoning even after it has been corrected (Chan, Jones, Hall Jamieson, & Albarracín, 2017; Ecker, Lewandowsky, & Apai, 2011b; Ecker, Lewandowsky, Swire, & Chang, 2011a; Ecker, Lewandowsky, & Tang, 2010; Gordon, Brooks, Quadflieg, Ecker, & Lewandowsky, 2017; Guillory & Geraci, 2016; Johnson & Seifert, 1994; Rich & Zaragoza, 2016; Wilkes & Leatherbarrow, 1988; for a review, see Lewandowsky, Ecker, Seifert, Schwarz, & Cook, 2012). Misinformation can have a lasting effect on people’s reasoning, even when they demonstrably remember that the information has been corrected (Johnson & Seifert, 1994) and are given prior warnings about the persistence of misinformation (Ecker et al., 2010).

In the experimental task used to study the continued-influence effect (CIE), participants are presented with a series of 10–15 sequentially presented statements describing an unfolding event. Target (mis)information that allows inferences to be drawn about the cause of the event is presented early in the sequence and is later corrected. Participants’ inferential reasoning and factual memory based on the event report are then assessed through a series of open-ended questions.

For example, in Johnson and Seifert (1994), participants read a story about a warehouse fire in which the target (mis)information implies that carelessly stored flammable materials (oil paint and gas cylinders) are a likely cause of the fire. Later in the story, some participants learned that no such materials had actually been stored in the warehouse, and therefore that they could not have caused the fire. The ensuing questionnaire included indirect inference questions (e.g., “what could have caused the explosions?”), as well as direct questions probing recall of the literal content of the story (e.g., “what was the cost of the damage done?”). The responses to inference questions were coded in order to measure whether the misinformation had been appropriately updated (no oil paint and gas cylinders were present in the warehouse). The responses were categorized according to whether they were consistent with the explanation implied by the target (mis)information^1^ (e.g., “exploding gas cylinders”) or were not (e.g., “electrical short circuit”).

In a typical CIE experiment, performance on a misinformation-followed-by-correction condition is usually compared to one or more baselines: a condition in which the misinformation is presented but is not then retracted (no-correction condition) or a condition in which the misinformation is never presented (no-misinformation condition). The former control condition allows for assessment of the retraction’s effectiveness; the latter arguably shows whether the correction reduces reference to misinformation to a level comparable to never having been exposed to the misinformation (but see below).

The key finding from CIE studies is that people continue to use the misinformation to answer the inference questions, even though it has been corrected. The most consistent pattern of findings is that references to previously corrected misinformation are elevated relative to a no-misinformation condition, and are either below, or in some cases indistinguishable from, references in the no-correction condition.

## Using open- and closed-ended questions online

With only a few exceptions (Guillory & Geraci, 2013, 2016; Rich & Zaragoza, 2016), research concerning reliance on misinformation has used open-ended questions administered in the lab (see Capella, Ophir, & Sutton, 2018, for an overview of approaches to measuring misinformation beliefs). There are several good reasons for using such questions, particularly on memory-based tasks that involve the comprehension or recall of previously studied text. First, the responses to open-ended questions are constructed rather than suggested by response options, and so avoid bias introduced by suggesting responses to participants. Second, open-ended questions also allow participants to give detailed responses about complex stimuli and permit a wide range of possible responses. Open-ended questions also resemble cued-recall tasks, which mostly depend on controlled retrieval processes (Jacoby, 1996) and provide limited retrieval cues (Graesser, Ozuru, & Sullins, 2010). These factors are particularly important for memory-based tasks wherein answering the questions requires the active generation of previously studied text (Ozuru, Briner, Kurby, & McNamara, 2013).

For Web-based testing, these advantages are balanced against the potential reduction in data quality when participants have to type extensive responses. The evidence concerning written responses is mixed. Grysman (2015) found that participants on the Amazon Mechanical Turk (AMT) wrote shorter self-report event narratives than did college participants completing online surveys, typing in the presence of a researcher, or giving verbal reports. Conversely, Behrend, Sharek, Meade, and Wiebe (2011) found no difference in the amounts written in free-text responses between university-based and AMT respondents.

A second potential effect concerns missing data: Participants have anecdotally reported to us that they did not enjoy typing open-ended responses. Open-ended questions could particularly discourage participants with lower levels of literacy or certain disabilities from expressing themselves in the written form, which could in turn increase selective dropout from some demographic groups (Berinsky, Margolis, & Sances, 2014). As well as losing whole participant datasets, open-ended questions in Web surveys could also result in more individual missing data points than closed-ended questions do (Reja, Manfreda, Hlebec, & Vehovar, 2003).

The alternative to using open-ended questions online is using closed-ended questions. These have many advantages, particularly in a context where there is less social pressure to perform diligently. However, response options can also inform participants about the researcher’s knowledge and expectations about the world and suggest a range of reasonable responses (Schwarz, Hippler, Deutsch, & Strack, 1985; Schwarz, Knauper, Hippler, Neumann, & Clark, 1991; Schwarz, Strack, Müller, & Chassein, 1988). There is also empirical evidence to suggest that open- and closed-end responses are supported by different cognitive (Frew, Whynes, & Wolstenholme, 2003; Frew, Wolstenholme, & Whynes, 2004) or memory (Khoe, Kroll, Yonelinas, Dobbins, & Knight, 2000; see Yonelinas, 2002, for a review) processes. A straightforward conversion of open- to closed-ended questions might therefore be impractical for testing novel scientific questions in a given domain.

The latter caveat may be particularly relevant for the CIE. Repeated statements are easier to process and are subsequently perceived as more truthful than new statements (Ecker, Lewandowsky, Swire, & Chang, 2011a; Fazio, Brashier, Payne, & Marsh, 2015; Moons, Mackie, & Garcia-Marques, 2009). Therefore, repeating misinformation in the response options could activate automatic (familiarity-based) rather than strategic (recollection-based) retrieval of studied text, which may not reflect how people reason about misinformation in the real world. Conversely, presenting corrections that explicitly repeat misinformation is more effective at reducing misinformation effects than is presenting corrections that avoid repetition (Ecker, Hogan, & Lewandowsky, 2017). As such, substituting closed-ended for open-ended questions might have unpredictable consequences.

## Overview of experiments

The overarching aim of the experiments reported here was to examine open- and closed-ended questions in Web-based memory and inference research. The more specific goals were (1) to establish whether a well-known experimental task that elicits responses with open-ended questions would replicate online, and (2) to explore the feasibility of converting open-ended questions to the type of closed-ended questions more typically seen online. To achieve these goals, two experiments were designed to replicate the CIE. Experiments 1A and 1B used the same experimental stimuli and subset of questions as in Johnson and Seifert (1994, Exp. 3A), wherein participants read a report about a warehouse fire and answered questions that assessed inferential reasoning about the story, factual accuracy, and the ability to recall the correction or control information (critical information). Experiments 1A and 2A employed standard open-ended measures, whereas a closed-ended analogue was used in Experiments 1B and 2B. Although they are reported as separate experiments, both Experiments 1A and 1B were run concurrently as one study, as were Experiments 2A and 2B, with participants being randomly allocated to each experiment, as well as to the experimental conditions within each experiment.

## Experiment 1A

### Method

#### Participants

A power analysis using the effect size observed in previous research using the same stimuli and experimental design (Johnson & Seifert, 1994; effect size obtained from the means in Exp. 3A) indicated that a minimum of 69 participants were required (*f* = 0.39, 1–*β* = .80, *α* = .05). In total, 78 US-based participants (50 males, 28 females; between 19 and 62 years of age, *M* = 31.78, *SD* = 10.10) were recruited via AMT. Only participants with a Human Intelligence Task (HIT) approval rating greater than or equal to 99% were recruited for the experiment, to ensure high-quality data without having to include attentional check questions (Peer, Vosgerau, & Acquisti, 2014). The participants were paid $2, and the median completion time was 11 min.

#### Stimuli and design

**Fig. 1:**
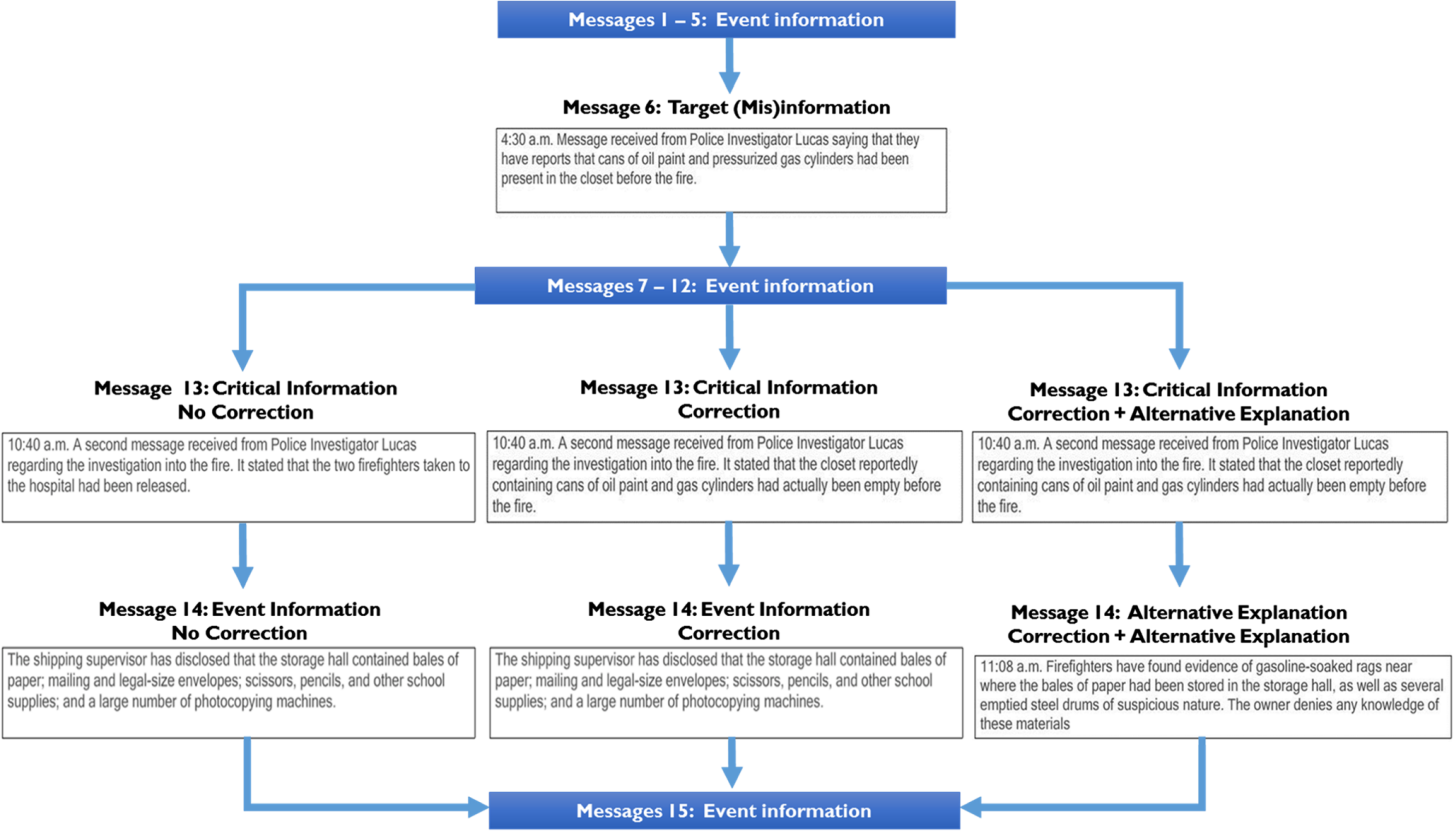
The *continued-influence effect* task: Messages 1–5 provide general information about the event, beginning with the fire being reported. The target (mis)information is presented at Message 6 and is then corrected, for correction and correction + alternative explanation groups, at Message 13. The correction + alternative explanation group then receive information providing a substitute account of the fire to “fill the gap” left by invalidating the misinformation. This condition usually leads to a robust reduction in reference to the misinformation

The experiment was programmed in Adobe Flash (Reimers & Stewart, 2007, 2015). Participants read one of three versions of a fictional news report about a warehouse fire, which consisted of 15 discrete messages. The stimuli were identical to those used in Johnson and Seifert (1994, Exp. 3A). Figure 1 illustrates how the message content was varied across the experimental conditions, as well as the message presentation format. The effect of the correction information on reference to the target (mis)information was assessed between groups; participants were randomly assigned to one of three experimental groups: no correction (*n* = 32), correction (*n* = 21), and alternative explanation (*n* = 25).

The target (mis)information, implying that carelessly stored oil paint and gas cylinders played a role in the fire, was presented at Message 6. This information was then corrected at Message 13 for the two conditions featuring a correction. Information implying that the fire was actually the result of arson (alternative explanation group) was presented at Message 14; the other two experimental groups merely learned that the storage hall contained stationery materials. The other messages provided further details about the incident and were identical in all three experimental conditions.

The questionnaire following the statements consisted of three question blocks: inference, factual, and critical information recall. The question order was randomized within the inference and factual blocks, but not in the correction recall block, in which the questions were presented in a predefined order: Inference questions (e.g., “What was a possible cause of the fumes”) were presented first, followed by factual questions (e.g., “What business was the firm in?”), and then critical information recall questions (e.g., “What was the point of the second message from Police Investigator Lucas?”).

There were three dependent measures: (1) reference to the target (mis)information in the inference questions, (2) factual recall, and (3) critical information recall. The first dependent measure assessed the extent to which the misinformation influenced interpretation of the news report, whereas the second assessed memory for the literal content of the report. The final measure specifically assessed understanding and accurate recall of the critical information that appeared at Message 13 (see Fig. 1). Although not all groups received a correction, the participants in all experimental groups were asked these questions so that the questions would not differ between the conditions. The stimuli were piloted on a small group of participants to check their average completion time and obtain feedback about the questionnaire. Following the pilot, the number of questions included in the inference and factual blocks was reduced from ten to six, because participants felt some questions were repetitive.

#### Procedure

Participants clicked on a link in AMT to enter the experimental site. After seeing details about the experiment, giving consent, and receiving detailed instructions, they were told that they would not be able to backtrack and that each message would appear for a minimum of 10 s before they could move on to the next message.

Immediately after reading the final statement, participants were informed that they would see a series of inference-based questions. They were told to type their responses in the text box provided, giving as much detail as necessary and writing in full sentences; that they should write at least 25 characters to be able to continue to the next question; and that they should answer questions on the basis of their understanding of the report and of industrial fires in general. After this they were informed that they would answer six factual questions, which then followed. Next, participants were instructed to answer the two critical information recall questions on the basis of what they remembered from the report. After completing the questionnaire, participants were asked to provide their sex, age, and highest level of education.

### Results

#### Coding of responses

The main dependent variable extracted from responses to the inference questions was “reference to target (mis)information.” References that explicitly stated, or strongly implied, that oil paint and gas cylinders caused or contributed to the fire were scored a 1; otherwise, responses were scored as 0. Table 1 shows an example of a response that was coded as a reference to target (mis)information and an example of a response that was not coded as such. There were several examples of references to flammable items that did not count as references to the corrected information. For example, stating that the fire spread quickly “Because there were a lot of flammable things in the shop” would not be counted as a reference to the corrected information, since there was no specific reference to gas, paint, liquids, substances, or the fact that they were (allegedly) in the closet. The maximum individual score across the inference questions was 6. The responses to factual questions were scored for accuracy; correct or partially correct responses were scored 1, and incorrect responses were scored 0. Again, the maximum factual score was 6. We also examined critical information recall, to check participants’ awareness of either the correction to the misinformation or the control message, computed using two questions that assessed awareness and accuracy for the critical information that appeared at Message 13. This meant that the correct response depended on correction information condition. For the participants in the no-correction group, the correct response was that the injured firefighters had been released from hospital, and for the two conditions featuring a correction, this was a correction of the target (mis)information.

**Table 1:** Example of response codings in Experiment 1

Question	Example of a Response Scored 1	Example of a Response Scored 0
Why did the fire spread so quickly?	Fire spread quickly due to gas cylinder explosion. Gas cylinders were stored inside the closet	The fire occurred in a stationery warehouse that housed envelopes and bales of paper that could easily ignite

#### Intercoder reliability

All participants’ responses to the inference, factual, and correction recall questions were independently coded by two trained coders. Interrater agreement was .88, and Cohen’s *Κ* = .76 ± .02, indicating a high level of agreement between coders; both measures are higher than the benchmark values of .7 and .6 (Krippendorff, 2012; Landis & Koch, 1977), respectively, and there was no systematic bias between raters, *χ*^2^ = 0.29, *p = .*59.

#### Inference responses

**Fig. 2:**
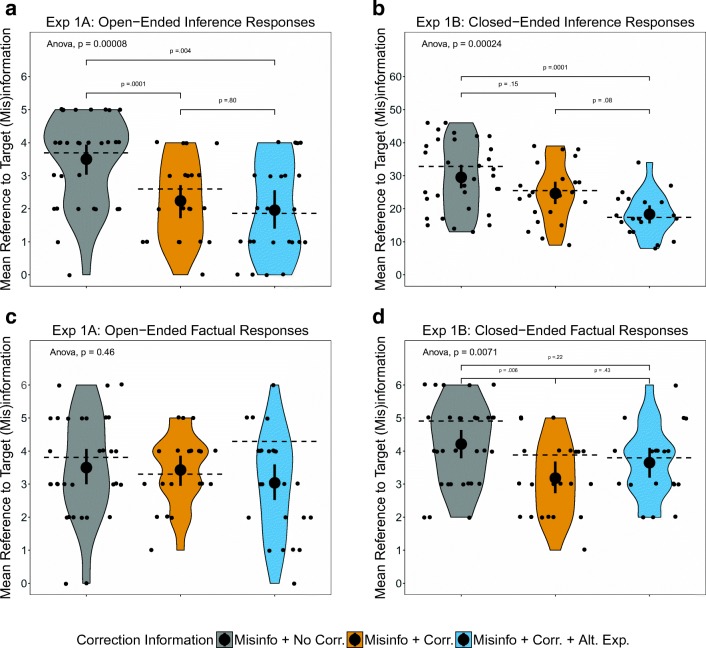
Effects of correction information on the numbers of (A) references to the target (mis)information in Experiment 1A, (B) references to the target misinformation in Experiment 1B, (C) accurately recalled facts in Experiment 1A, and (D) accurately recalled facts in Experiment 1B. Error bars represent 95% confidence intervals of the means. The brackets represent Dunnett’s multiple comparison tests (which account for unequal group sizes) for significant omnibus tests. The dashed lines represent the means after excluding participants who did not recall the critical information (i.e., scored 0 on the first critical information recall question asking what the point of the second message from Police Investigator Lucas was)

The overall effect of the correction information on references to the target (mis)information was significant, *F*(2, 75) = 10.73, *p* < .001, *η*_p_^2^ = .22 [.07, .36]. Dunnett multiple comparison tests (shown in panel A of Fig. 2) revealed that a correction or a correction with an alternative explanation significantly reduced reference to the target (mis)information in response to the inference questions.

A Bayesian analysis using the BayesFactor package in R and default priors (Morey & Rouder, 2015) was performed to examine the relative predictive success of the comparisons between conditions. The BF_10_ for the first comparison 28.93, indicating strong evidence (Lee & Wagenmakers, 2014) in favor of the alternative that there was a difference between the no correction and correction groups. The BF_10_ for the comparison between the no-correction and alternative-explanation groups was 209.03, again indicating very strong evidence in favor of the alternative. The BF_10_ was 0.36 for the final comparison between the correction and alternative-explanation groups, indicating anecdotal evidence in favor of the null.

The Bayes factor analysis was mostly consistent with the *p* values and effect sizes. Both conditions featuring a correction led to a decrease in references to the target (mis)information, but the data for the two conditions featuring a correction cannot distinguish between the null hypothesis and previous findings (i.e., that an alternative explanation substantially reduces reference to misinformation, as compared to a correction alone).

#### Factual responses

Factual responses were examined to establish whether the differences in references to the (mis)information could be explained by memory for the literal content of the report. Overall, participants accurately recalled similar numbers of correct details across the correction information conditions (Fig. 2C), and the omnibus test was not significant, *F*(2, 75) = 0.78, *p* = .46, *η*_p_^2^ = .02.

#### Response quality

Participants were required to write a minimum of 25 characters in response to the questions. The number of characters written was examined as a measure of response quality. Participants wrote between 36% and 64% more, on average, than the minimum required 25 characters in response to the inference (*M* = 69.45, *SD* = 40.49), factual (*M* = 39.09, *SD* = 15.85), and critical information recall (*M* = 66.72, *SD* = 42.76) questions. There was—unsurprisingly—a positive correlation between time taken to complete the study and number of characters written, *r*(76) = .31, *p* = .007.

## Experiment 1B

In Experiment 1B we examined the feasibility of converting open-ended questions to a comparable closed-ended form.

### Method

#### Participants

Seventy-five US-based (46 male, 29 female; between 18 and 61 years of age, *M* = 34.31, *SD* = 10.54) participants were recruited from AMT. The participants were paid $2; the median completion time was 9 min.

#### Design, stimuli, and procedure

**Fig. 3:**
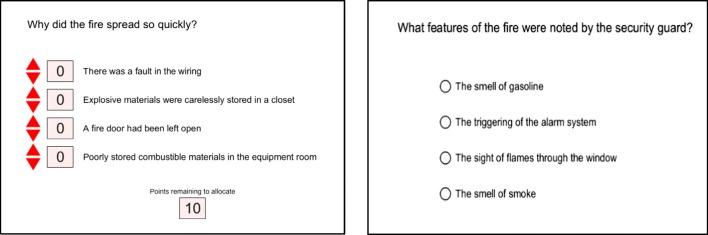
Screenshots of how the inference (left) and factual (right) questions and response options were presented to participants. Participants used the red arrow features to allocate points to the response alternatives in response to the inference questions. The factual questions were answered by selecting the “correct” option based on the information in the report

Experiment 1B used the same story/newsfeed stimuli and high-level design as Experiment 1A; participants were randomly assigned to one of three experimental conditions: no correction (*n* = 33), correction (*n* = 22), or alternative explanation (*n* = 20). The only difference between the experiments was that closed-ended questions were used in the subsequent questionnaire. Figure 3 shows how participants had to respond to inference and factual questions. For the inferential questions, points were allocated to response alternatives that corresponded to four possible explanations. For example, when answering the question “What could have caused the explosions?,” participants could allocate points to a misinformation-consistent option (e.g., “Fire came in contact with compressed gas cylinders”), an alternative-explanation-consistent option (e.g., “Steel drums filled with liquid accelerants”), an option that was plausible given the story details but that was not explicitly stated (e.g., “Volatile compounds in photocopiers caught on fire”), or an option that was inconsistent with the story details (e.g., “Cooking equipment caught on fire”).

The response options were chosen in this way to give participants the opportunity to provide more nuanced responses than would be possible using multiple-choice or true/false alternatives. This approach allowed the participants who were presented with misinformation and then a correction to choose an explanation that was consistent with the story but did not make use of the target (mis)information. If the CIE were observed in response to closed-ended questions, then the number of points allocated to misinformation-consistent options in the conditions featuring a correction should be non-zero. The accuracy on factual questions was measured using four-alternative forced choice multiple-choice questions, in which participants responded by choosing the correct answer from a set of four possible options. The order of presentation for the response alternatives for inference and factual questions was randomized across participants. The critical informatin  recall questions were open-ended, and participants gave free-text responses in the same manner as Experiment 1A.

### Results

Individual inference, factual, and critical information recall scores (an analysis of the critical information recall responses is shown in the additional analyses in the supplemental materials) were calculated for each participant. Since the maximum number of points that could be allocated to a given option explanation theme for each question was 10, the maximum inference score for an individual participant was 60. The maximum factual score was 6, and the maximum critical information  recall score was 2. Critical information recall questions were open-ended, and responses were coded using the same criteria as in Experiment 1A.

#### Inference responses

A one-way analysis of variance (ANOVA) on reference to the target (mis)information revealed a significant effect of correction information, *F*(2, 72) = 9.39, *p* < .001, *η*_p_^2^ = .21 [.05, .35]. Overall, the pattern of results for reference to the target (mis)information in response to closed-ended questions was very similar to that in Experiment 1A (Fig. 2B). Although a correction with an alternative explanation significantly reduced reference to the target (mis)information, a correction on its own did not. The difference between the two conditions featuring a correction was also not significant.

The BF_10_ was 1.02 for the first comparison, between the no-correction and correction groups, indicating anecdotal evidence in favor of the alternative, or arbitrary evidence for either hypothesis. The BF_10_ was 250.81 for the second comparison, between the no-correction and alternative-explanation groups, indicating strong evidence for the alternative. The BF_10_ was 4.22 for the final comparison, indicating substantial evidence in favor of the alternative.

The Bayes factor analysis was mostly consistent with the *p* values and effect sizes, except that the Bayes factor for the comparison between the correction and alternative-explanation conditions suggested an effect, whereas the *p* value did not.

#### Factual responses

Analysis of the factual scores indicated a significant difference between the correction information groups, *F*(2, 72) = 5.30, *p* = .007, *η*_p_^2^ = .13 [.01, .26]. Figure 2D shows that the average number of factually correct details recalled from the report was significantly lower in the correction condition than in the no-correction group but not than in the alternative-explanation group. The poorer overall performance on factual questions for the correction group was mainly attributable to incorrect responses to two questions. The first of these questions asked about the contents of the closet that had reportedly contained flammable materials, before the fire; the second asked about the time the fire was put out. Only a third (23% in the correction and 25% in the alternative-explanation group) answered the question about the contents of the closet correctly (i.e., that the storeroom was empty before the fire), whereas 86% of the no-correction group correctly responded that oil paint and gas cylinders were in the storeroom before the fire. This is perhaps unsurprising: The correct answer for the no-correction condition (“paint and gas cylinders”) was more salient and unambiguous than the correct answer for the other two conditions (“The storage closet was empty before the fire”).

### Discussion

The results for Experiments 1A and 1B suggest that both open- and closed-ended questions can successfully be used in online experiments with AMT to measure differences in references to misinformation in a standard continued-influence experiment. There was a clear CIE of misinformation in all conditions of both experiments—a correction reduced, but did not come near eliminating, reference to misinformation in inference questions. In both experiments, references to target (mis)information were significantly lower in the correction + alternative than in the no-correction condition, with the correction condition lying between those two extremes (see Fig. 2A and B). Although the patterns of significant results were slightly different (correction condition was significantly below no correction in Exp. 1A but not in Exp. 1B), this is consistent the variability seen across experiments using the CIE, in that some researchers have found a reduction in references to (mis)information following a correction (Connor Desai & Reimers, 2017; Ecker, Lewandowsky, & Apai, 2011b; Ecker et al., 2010), but others have found no significant reduction (Johnson & Seifert, 1994).

With regard to motivation, we found that the vast majority of participants wrote reasonable responses to the open-ended questions. The answers were of a considerable length for the question, with participants usually typing substantially more than the minimum number of characters required. We found that the absolute numbers of references to the misinformation were comparable to those found in existing studies. That said, the open-ended questions had to be coded by hand, and for participants the median completion time was 18% longer in Experiment 1A (11 min) than in Experiment 1B (9 min). This disparity in completion times only serves to emphasize that using closed-ended questions streamlines the data collection process relative to open-ended questions.

Taken as a whole, these findings show that reasonably complex experimental tasks that traditionally require participants to construct written responses can be implemented online using either the same type of open-ended questions or comparable closed-ended questions.

## Rationale for Experiments 2A and 2B

The results of Experiments 1A and 1B are promising with regard to using open-ended questions in online research in general, and to examining phenomena such as the CIE specifically. However, they have some limitations. The most salient limitation was the sample size. Although the numbers of participants in the different conditions were comparable to those in many lab-based studies of the CIE, the sample size was nonetheless small. One of the advantages of using Web-based procedures is that it is relatively straightforward to recruit large numbers of participants, so in Experiments 2A and 2B we replicated the key conditions of the previous studies with twice as many participants. We also preregistered the method, directional hypotheses, and analysis plan (including planned analyses, data stopping rule, and exclusion criteria) prior to data collection; this information can be found at https://osf.io/cte3g/.

We also used this opportunity to include a second baseline condition. Several CIE experiments have included control conditions in some form that makes it possible to see whether references to the cause suggested by the misinformation following its correction are not only greater than zero, but greater than the references to the same cause if the misinformation is never presented. In this study we did not believe that such a condition would be very informative, because the strictness of the coding criteria meant that it would be unlikely that participants would spontaneously suggest paint or gas cylinders as contributing to the fire.^2^

Instead, Experiments 2A and 2B included a more directly comparable control group for whom a correction was presented without the initial target (mis)information. According to the mental-model-updating account of the CIE, event information is integrated into a mental model that is updated when new information becomes available. Corrections may be poorly encoded or retrieved because they threaten the model’s internal coherence (Ecker et al., 2010; Johnson & Seifert, 1994; Johnson-Laird, 1980). If the CIE arises because of a mental-model-updating failure, then presenting the misinformation only as part of a correction should not result in a CIE, because there would not be an opportunity to develop a mental model involving the misinformation. On the other hand, participants might continue to refer to the misinformation for more superficial reasons: If the cause presented in the misinformation were available in memory and recalled without the context of its being corrected, then presenting the misinformation as part of the correction should lead to a CIE comparable to those in other conditions.

In these experiments, we repeated the no-correction and correction conditions from Experiments 1A and 1B. In place of the correction + alternative condition, however, we had the no-mention condition, which was the same as the correction condition except that we replaced the target (mis)information with a filler statement (“Message 6—4:30 a.m. Message received from Police Investigator Lucas saying that they have urged local residents to keep their windows and doors shut”). The wording of the correction message for this condition stated that “a closet reportedly containing cans of oil paint and gas cylinders had actually been empty before the fire” rather than referring simply to “the closet,” so that the participants would not think they had missed some earlier information.

Beyond this, the general setup for Experiments 2A and 2B was the same as that for Experiments 1A and 1B, except in the following respects: We included an instruction check (which appeared immediately after the initial instructions and immediately before the warehouse fire report was presented) that tested participants’ comprehension of the instructions via three multiple-choice questions. Participants were not excluded because of this check, but they were not allowed to proceed to the main experiment until they had answered all three questions correctly, consistent with Crump et al.’s (2013) recommendations. Because Adobe Flash, which we had used for Experiments 1A and 1B, is being deprecated and is increasingly hard to use for Web-based research, we implemented Experiments 2A and 2B using Qualtrics, which led to some superficial changes in the implementation. Most notable was that the point-allocation method for closed-ended inference questions required participants to type numbers of points to allocate, rather than adjusting the values using buttons.

The sample size was also doubled in this second set of experiments.

## Experiment 2A

### Method

#### Participants

In all, 157 US- and UK-based participants (91 male, 66 female; between 18 and 64 years of age, *M* = 33.98, *SD* = 10.57) were recruited using AMT.^3^ The median completion time was 16 min and participants, and were paid $1.25.^4^

#### Design and procedure

Participants were randomly assigned to one of three experimental conditions: misinformation + no correction (*n* = 52), misinformation + correction (*n* = 52), or no misinformation + correction (*n* = 53).

### Results

#### Intercoder reliability

Participants’ responses to the inference, factual, and critical information recall 5 questions were coded by one trained coder, and 10% (*n* = 16) of the responses were independently coded by a second trained coder. The interrater agreement was 1 and Cohen’s *K* = 1 ± 0, indicating, surprisingly, perfect agreement between the coders.

#### Inference responses

**Fig. 4:**
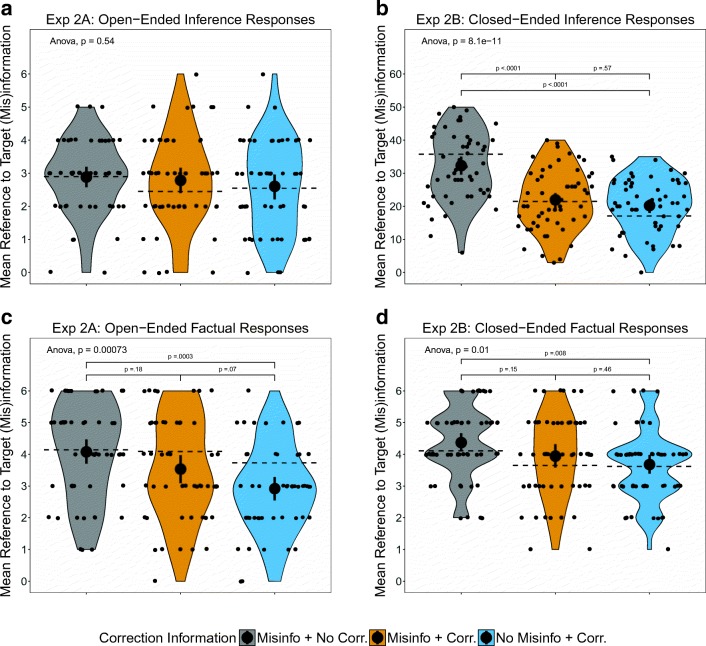
Effects of correction information on the numbers of (A) references to the target (mis)information in Experiment 2A, (B) references to the target (mis)information in Experiment 2B, (C) accurately recalled facts in Experiment 2A, and (D) accurately recalled facts in Experiment 2B. Error bars represent 95% confidence intervals of the means. The brackets represent Tukey multiple comparison tests when the omnibus test was significant. The dashed lines represent the means for the restricted sample of participants who did not answer the first critical information recall question correctly

Participants produced similar numbers of references to the target (mis)information across correction information conditions (Fig. 4A), and the omnibus test was not significant, *F*(2, 154) = 0.62, *p = .*54, *η*_p_^2^ = .01 [.00, .05]. Unlike in Experiment 1A, a correction did not significantly reduce the number of references to the target (mis)information relative to a control group who did not receive a correction. Moreover, participants who were not presented with the initial misinformation but did receive a correction message, made a number of misinformation references similar to those for participants who were first exposed to the misinformation.

#### Factual responses

Participants’ ability to accurately recall details from the report differed across correction information conditions (Fig. 4C), *F*(2, 154) = 8.12, *p* < .001, *η*_p_^2^ = .10 [.02, .18]. Tukey’s test for multiple comparisons revealed that the group who received a correction without the initial misinformation recalled significantly fewer details from the report than did the group who saw the uncorrected misinformation, but the other differences were nonsignificant, *p*s > .05.

#### Response quality

Participants wrote between 48% and 69% more, on average, than the minimum of 25 required characters in response to the inference (*M* = 80.76, *SD* = 56.38), factual (*M* = 48.15, *SD* = 24.86), and critical information recall (*M* = 75.56, *SD* = 47.05) questions. We found a positive correlation between the time taken to complete the study and the number of characters written, *r*(155) = .34, *p* < .0001, showing that the participants who took longer wrote more.

## Experiment 2B

### Method

#### Participants

A total of 166 US- and UK-based participants (100 male, 66 female; between 18 and 62 years of age, *M* = 35.04, *SD* = 10.36) were recruited using AMT.^6^ Participants were paid $1.25; their median completion time was 13 min.

#### Design and procedure

Experiment 2B used the same high-level design and procedure as Experiment 2A. The responses were closed-ended and made in the same way as in Experiment 1B. Participants were randomly assigned to one of three experimental conditions: misinformation + no correction (*n* = 54), misinformation + correction (*n* = 56), or no misinformation + correction (*n* = 56).

### Results

#### Inference responses

We found a significant effect of correction information on references to the target (mis)information for closed-ended measures (Fig. 4B), *F*(2, 163) = 26.90, *p* < .001, *η*_p_^2^ = .25 [.14, .35]. Tukey-adjusted multiple comparisons further revealed that the group exposed to misinformation and its correction, and the group who saw only the correction without the initial misinformation, made significantly fewer references to the target (mis)information than did the uncorrected misinformation condition. The two groups who received correction information did not differ significantly.

#### Factual responses

Participants’ responses to the factual questions also showed a significant effect of correction information condition (Fig. 4D), *F*(2, 163) = 4.70, *p* = .01, *η*_p_^2^ = .05 [.00, .13]. Tukey’s tests revealed that the factual responses from participants in the condition featuring a correction without the initial misinformation were significantly lower than those from the group who saw uncorrected misinformation. The other differences were not significant (*p*s > .1). A closer inspection of the individual answers revealed that incorrect responses for the no misinformation + correction group were mainly attributable to the question asking about the contents of the closet before the fire.

#### Dropout analysis

Of the 375 people who started the study, only 323 fully completed it (dropout rate 13%). Of those who completed the study, four (1.23%) were excluded prior to the analysis because they gave nonsense open-ended responses (e.g., “21st century fox, the biggest movie in theatre”). The majority of participants who dropped out did so immediately after entering their worker ID and before being assigned to a condition (41%). Of the remaining dropout participants who were assigned to a condition, 27% were assigned to one of the open-ended conditions and dropped out during the first question block. A further 16% were assigned to one of the closed-ended conditions and dropped out when asked to answer the open-ended critical information recall questions. The remaining 14% were assigned to a closed-ended condition and dropped out as soon as they reached the first question block. The dropout breakdown suggests that many people dropped out because they were unhappy about having to give open-ended responses. Some participants who were assigned to the closed-ended conditions dropped out when faced with open-ended questions, despite the fact that the progress bar showed that they had almost completed the study.

### Discussion

Experiments 2A and 2B again showed clear evidence of a CIE. As in Experiments 1A and 1B, participants continued to refer to the misinformation after it had been corrected. Also consistent with the previous two experiments, the effects of a correction differed slightly across conditions. This time the reduction in references to the (mis)information was significant for the closed-ended questions, but not for the open-ended questions. As we noted earlier, this is consistent with findings that a correction sometimes reduces references to misinformation relative to no correction, and sometimes it does not (Connor Desai & Reimers, 2017; Ecker et al., 2010).

Experiments 2A and 2B also included a novel control condition in which participants were not exposed to the initial misinformation but were exposed to its correction. Contrary to expectations, the new condition resulted in a number of references to the target (mis)information that was statistically equivalent to that in the group who were exposed to both the misinformation and its correction. This finding suggests that the CIE might not reflect a model-updating failure, but rather a decontextualized recall process.

## General discussion

In four experiments we examined the feasibility of collecting data on the CIE online, comparing the efficacy of using traditional open-ended questions versus adapting the task to use closed-ended questions. For both types of elicitation procedures, we observed clear CIEs: Following an unambiguous correction of earlier misinformation, participants continued to refer to the misinformation when answering inferential questions. As such, these studies provide clear evidence that both open-ended and closed-ended questions can be used in online experiments.

### The continued-influence effect

Across all four studies we found that participants continued to use misinformation that had been subsequently corrected. This occurred even though a majority of participants recalled the correction. We found mixed results when examining whether a correction had any effect at all in reducing references to misinformation. Experiments using similar designs have both found (Ecker, Lewandowsky, & Apai, 2011b; Ecker et al., 2010) and failed to find (Johnson & Seifert, 1994) an effect of a correction. Overall, we found limited evidence for an effect of a correction for the open-ended questions, but substantial evidence for an effect of a correction using closed-ended questions. For open-ended questions, it appears that any effect of a correction on reference to misinformation—at least using this scenario—is relatively small, and would be hard to detect consistently using the small sample sizes that have traditionally been used in this area. This may explain the variability in findings in the literature.

A correction with an alternative explanation appeared (at least numerically) to be more effective in reducing reliance on misinformation than a correction alone. Furthermore, given that Experiment 1B’s results were actually more consistent with the original finding (Johnson & Seifert, 1994), the differences between past and present work are most likely unsystematic and therefore unrelated to the online testing environment or question type.

Finally, with regard to the main results, in Experiments 2A and 2B we found using a novel condition, that misinformation that was only presented as part of a correction had as much of a continuing influence effect as misinformation presented early in a series of statements and only later corrected. This has both theoretical and practical implications. Theoretically, it suggests that—under some circumstances—the CIE may not be the result of participants’ unwillingness to give up an existing mental model without an alternative explanation (Ecker, Lewandowsky, & Apai, 2011b; Ecker, Lewandowsky, Swire, & Chang, 2011a; Johnson & Seifert, 1994). Instead, it might be that participants search their memory for possible causes when asked inferential questions, but fail to retrieve the information correcting the misinformation.

### Open- and closed-ended questions and the CIE

The pattern of results in response to inference questions was qualitatively very similar across both open and closed ended questions. This finding is particularly interesting in light of the fact that responses to open and closed questions might be supported by different underlying retrieval processes (Fisher, Brewer, & Mitchell, 2009; Ozuru et al., 2013; Shapiro, 2006). Crucially, the response options used in Experiments 1B and 2B required participants to make a more considered judgment than multiple-choice or yes/no questions, which may have encouraged recall rather than a familiarity-based heuristic. It is also interesting that participants still referred to the incorrect misinformation despite the fact that another response option was consistent with the report, although this was not explicitly stated.

Another important observation was that we found an effect of correction information on responses to closed-ended factual questions, but not to open questions. The difference between conditions is significant, because it was partly attributable to a question that probed participants’ verbatim memory of the correction. Many participants in both conditions featuring a correction answered this question incorrectly, despite the fact that the options clearly distinguished between the correct and incorrect answers, given what participants had read. This question asked what the contents of the closet were before the fire, so it not hard to see why participants who continued to rely on the misinformation might have answer this question incorrectly. The fact that there were differences between the conditions highlights the importance of carefully wording questions and response options in order to avoid bias.

It is also worth noting that floor effects were not observed (i.e., the misinformation was still influential for both groups that received a correction), despite the fact that the present study did not include a distractor task and that participants answered the inference questions directly after reading the news report (and so, theoretically, should have had better memory for the report details).

A brief note on the use of closed-ended questions and response alternatives: There is the possibility that presenting a closed list of options reminded participants of the arson materials explanation and inhibited responses consistent with the oil paint and gas cylinders explanation. Also, the closed list of options that repeated the misinformation could have increased its familiarity, making it more likely to be accepted as true (e.g., Ecker, Lewandowsky, Swire, & Chang, 2011a). For the group that received a simple correction, the other options had not been explicitly stated in the story. These participants may not have fully read or understood the question block instructions, and therefore perceived the task as choosing the option that had appeared in the story, irrespective of the correction. In contrast, the participants in the alternative-explanation group were able to better detect the discrepancy between the misinformation and its correction, because of the option alluding to arson materials. Although the response alternatives provided a plausible response that was consistent with the details of the fire story, none of the options made it possible to rule out that participants just did not consider the correction when responding. The response alternatives provided forced the participants to choose one from among four explanations, which may not have reflected their understanding of the event, but nonetheless was the option that was most consistent with what they had read. This explanation is also consistent with previous studies showing that the response options chosen by the researcher can be used by the participants to infer which information the participant considers relevant (Schwarz et al., 1985; Schwarz et al., 1991).

### Open- and closed-ended questions in Web-based research

As well as looking directly at the CIE, we also examined the extent to which participants recruited via Amazon Mechanical Turk could provide high-quality data from open-ended questions. We found high levels of diligence—participants typed much more than was required in order to give full answers to the questions, they spent more time reading statements than was required, and—with a small number of exceptions—they engaged well with the task and attempted to answer the questions set.

We found that dropout did increase, however, when participants had to give open-ended responses. This may suggest that some participants dislike typing open-ended responses, to the extent that they choose not to participate. (It could be that participants find it too much effort, or that they do not feel confident giving written answers, or that it feels more personal having to type an answer oneself.) Alternatively, it may be that some participants, because of the device they were using, would struggle to provide open-ended responses, and so dropped out when faced with open-ended questions. Either way, it is striking that we had over 4% of the participants in Experiment 2B who read all the statements and gave answers to all the closed-ended questions, but then dropped out when asked to type their responses to the final two critical information recall questions. There are ethical implications of having participants spend 10 min on a task before dropping out, so the requirement for typed answers should be presented prominently before participants begin the experiment.

We found that participants’ recall of the correction for the misinformation was worse than in previous lab-based studies. We found that only a little over half of participants across the conditions in our study correctly reported the correction when prompted. This figure is poor when compared to the figures of 95% (correction) and 75% (alternative explanation) found in Johnson and Seifert’s (1994, Exp. 3A) laboratory-based experiment. It is possible that this was the result of poor attention and recall of the correction, but we believe it was more likely a response issue, in which participants retained the information but did not realize that they were being asked to report it when asked whether they were aware of any inconsistencies or corrections. (In other unpublished research, we have found that simply labeling the relevant statement “Correction:” greatly increased participants’ reference to it when asked about any corrections.) Although this did not affect the CIE, in future research we would recommend making the instructions for the critical information recall questions particularly clear and explicit. This advice would, we imagine, generalize to any questions that might be ambiguous and would require a precise answer.

In choosing whether to use open-ended questions or to adapt them to closed-ended questions for use online, there are several pros and cons to weigh up. Open-ended questions allow for a consistency of methodology with traditional lab-based approaches—meaning there is no risk of participants switching to using different strategies or processes, as they might with closed-ended questions. We have shown that participants generally engage well and give good responses to open-ended questions. It is also much easier to spot and exclude participants who respond with minimal effort, since their written answers tend to be nonsense or copied and pasted from elsewhere. For closed-ended responses, attention or consistency checks or other measures of participant engagement are more likely to be necessary. That said, closed-ended questions are, we have found, substantially faster to complete, meaning that researchers on a budget could test more participants or ask more questions; such questions require no time to manually code; participants are less likely to drop out with them; and—at least in the area of research used here—they provide results comparable to those from open-ended questions.

### Conclusion

In conclusion, the *continued-influence effect* can be added to the existing list of psychological findings that have been successfully replicated online. Data obtained online are of sufficiently high quality to allow examining original research questions and are comparable to data collected in the laboratory. Furthermore, the influence of misinformation can be examined using closed-ended questions with direct choices between options. Nevertheless, as with any methodological tool, researchers should proceed with caution and ensure that sufficient piloting is conducted prior to extensive testing. More generally, the research reported here suggests that open-ended written responses can be collected via the Web and Amazon Mechanical Turk.

#### Author note

We thank Cassandra Springate for help with coding the data.

## Electronic supplementary material

ESM 1 This analysis differed from the preregistered confirmatory analysis. We planned to compare the conditions using t-tests but instead used chi-squared tests for the following reason. The second question (“Were you aware of any corrections or contradictions in the story you read”) was only relevant to the conditions featuring initial misinformation and its correction. We wanted to be able to compare all three conditions so only used the first question which was applicable to all three conditions. Accordingly, we used chi-square tests to test for dependence between correction information condition and recall of critical information. (DOCX 64 kb)

## Footnotes

1 We use the term *(mis)information* throughout to refer to the original statement presented early in a CIE study that is later corrected. We parenthesize the *(mis)* because in some control conditions the information is not corrected, meaning that it cannot be considered misinformation from those participants’ perspective.

2 There was also a conceptual issue concerning whether references to the cause presented in the misinformation should be compared across correction and no-mention conditions. In the former case, the correction ruled out the cause; in the latter, the cause would still be possible.

3 Three of the participants were recruited from Prolific Academic. Data was collected from 159 participants but two of the participants were excluded because they gave nonsense answers to the questions (e.g., “because the wind is blow, love is fall, I think it is very interesting”).

4 The modal completion time in Experiments 1 and 2 was below 10 min, so the fee was reduced so that participants were paid the equivalent of the federal minimum wage in the US ($7.25).

5 Critical information recall is referred to as correction recall in the preregistration document submitted for the second set of studies reported. We changed the name of this variable to reflect the fact that a correction was not presented in the no-correction condition.

6 The recruited number of participants differed from stopping rule specified in the preregistration. In total, 168 participants were recruited for the closed-ended condition, due to an error. Ultimately we decided to include the extra participants in the analysis rather than exclude their data. However, the responses from two participants were excluded: one because the participant took the HIT twice, and another because the participant provided nonsense answers to the open-ended questions at the end of the study.
