# Supplementary material for: Comparing the use of open and closed questions for Web-based measures of the continued-influence effect
Source: Behav Res Methods. 2018 Jun 25;51(3):1426–40. doi: 10.3758/s13428-018-1066-z (PMC6538818; doi:10.3758/s13428-018-1066-z)
Supplement: Supplementary file 1 — This analysis differed from the preregistered confirmatory analysis. We planned to compare the conditions using t-tests but instead used chi-squared tests for the following reason. The second question (“Were you aware of any corrections or contradictions in the story you read”) was only relevant to the conditions featuring initial misinformation and its correction. We wanted to be able to compare all three conditions so only used the first question which was applicable to all three conditions. Accordingly, we used chi-square tests to test for dependence between correction information condition and recall of critical information. (DOCX 64 kb) [file 13428_2018_1066_MOESM1_ESM.docx]

# Supplementary Materials

## Questions and closed-ended response options

**Inference Questions**

1. Why did the fire spread so quickly?

1. Burning paint may have spilled over a large area
2. Flammable materials could have been deliberately soaked in gasoline
3. There could have been large amounts of paper throughout the building
4. The kitchen door may have been left open

2. What was the possible cause of the fumes?

1. Oil-based paint
2. Gasoline
3. Paper and cardboard
4. Cooking oil

3. What aspect of the fire might the police want to continue investigating?

1. Dangerously flammable materials were stored carelessly
2. The presence of items of a suspicious nature
3. Unaddressed fire code violations
4. Fire not adequately prevented by open fire door

4. What could have caused the explosions?

1. Fire came in contact with compressed gas cylinders
2. Steel drums filled with liquid accelerants
3. Volatile compounds in photocopiers caught on fire
4. Cooking equipment caught on fire

5. Where was the probable location of the explosions?

1. The storage closet
2. The storage hall
3. The display room
4. The kitchen

6. What was the most likely overall cause of the fire?

1. Flammable liquids and gases not stored properly
2. Someone deliberately set fire to the property
3. The owner had allowed paper and cardboard to be left lying around
4. The cooker in the kitchen was left on

**Factual Questions**

1. Where on the premises was the fire located?
   1. In a closet off the main storage hall
   2. In the storage hall
   3. In the owner’s office
   4. In a supply room, next to the storage hall
2. What features of the fire were noted by the security guard?
   1. The smell of smoke
   2. The smell of gasoline
   3. The triggering of the alarm system
   4. The sight of flames through the window
3. What business was the firm in?
4. Wholesale stationery
5. Toy manufacturer
6. Electrical supplies producer
7. Book printing services

4. What was present in the closet before the fire?

1. Cans of oil paint and pressurised gas cylinders
2. The storage closet was empty before the fire
3. Printer cartridges and toners
4. The worker’s uniforms

5. What was the cost of the damage done?

1. Hundreds of thousands of dollars
2. Millions of dollars
3. Hundreds of dollars
4. Tens of thousands of dollars
5. When was the fire eventually put out?
6. 11.30 a.m.
7. 11.08 a.m.
8. 6.30 a.m.
9. 12.00 p.m.

## Additional Analyses

In order to make comparisons between conditions, responses to the question probing recall of critical information that appeared at Message 13 (i.e., either a correction or control message) were analysed. This analysis differed from the preregistered confirmatory analysis. The second question was only relevant to the conditions featuring initial misinformation and its correction so were not analysed. Chi-square tests tested dependence between correction information condition and recall of critical information.

**Effect of correction information condition on critical information recall responses (Experiment 1A)**

Relative frequencies did not significantly differ, $\boldsymbol{\chi}^{\boldsymbol{2}}$ (2) = 3.12, *p* = .21. Accurate recall of critical information occurred at rate of 50% for the no correction group, 48% for the correction group, and 28% for the alternative explanation group.

**Effect of correction information condition on critical information recall responses (Experiment 1B)**

Relative frequencies did not significantly differ, $\boldsymbol{\chi}^{\boldsymbol{2}}$ (2) = 0.67, *p* = .72. Accurate recall of critical information occurred at rate of 33% for the no correction group, 36% for the correction group, and 25% for the alternative explanation group.

**Effect of correction information condition on critical information recall responses (Experiment 2A)**

Relative frequencies were significantly different, $\boldsymbol{\chi}^{\boldsymbol{2}}$ (2) = 13.73, *p* = .001. The no correction group recalled critical information at a rate of 56% the correction group accurately recalled critical information at a rate of 42% and the no misinformation group at a rate of 21%.

**Effect of correction information condition on critical information recall responses (Experiment 2B)**

Relative critical information recall frequencies were significantly different, $\boldsymbol{\chi}^{\boldsymbol{2}}$ (2) = 21.09, *p* < .001. The no correction group recalled critical information at a rate of 50% the correction group accurately recalled critical information at a rate of 66% and the no misinformation group at a rate of 22%.
